# Physicochemical characterization of the recombinant lectin scytovirin and microbicidal activity of the SD1 domain produced in rice against HIV-1

**DOI:** 10.1007/s00299-022-02834-5

**Published:** 2022-02-18

**Authors:** Victoria Armario-Najera, Amaya Blanco-Perera, Shilpa R. Shenoy, Yi Sun, Silvia Marfil, Jordana Muñoz-Basagoiti, Daniel Perez-Zsolt, Julià Blanco, Nuria Izquierdo-Useros, Teresa Capell, Barry R. O’Keefe, Paul Christou

**Affiliations:** 1 Department of Plant Production and Forestry Science, School of Agrifood and Forestry Science and Engineering, University of Lleida-Agrotecnio CERCA Center, 25198 Lleida, Spain; 2 Frederick National Laboratory for Cancer Research, Leidos Biomedical Research Inc., Frederick, MD 21702 USA; 3 Molecular Targets Program, Center for Cancer Research, National Cancer Institute, NIH, Frederick, MD USA; 4 IrsiCaixa AIDS Research Institute, 08916 Badalona, Spain; 5 Germans Trias i Pujol Research Institute (IGTP), Can Ruti Campus, 08916 Badalona, Spain; 6 Chair of AIDS and Related Diseases, University of Vic-Central University of Catalonia, 08500 Vic, Barcelona Spain; 7 Natural Products Branch, Developmental Therapeutics Program, Division of Cancer Treatment and Diagnosis, National Cancer Institute, NIH, Frederick, MD USA; 8 Catalan Institute for Research and Advanced Studies (ICREA), 08010 Barcelona, Spain

**Keywords:** Antiviral lectins, HIV, Scytovirin, Transgenic plant

## Abstract

**Key message:**

**Rice-produced SD1 retains its physicochemical properties and provides efficient pre-exposure HIV-1 prophylaxis against infection in vitro.**

**Abstract:**

Scytovirin (SVN) is an HIV-neutralizing lectin that features two structural domains (SD1 and SD2) that bind to HIV-1 envelope glycoproteins. We expressed SD1 in rice seeds as a potential large-scale production platform and confirmed that rice-derived SD1 binds the HIV-1 envelope glycoprotein gp120 in vitro. We analyzed the thermodynamic properties of SD1 compared to full-size SVN (produced in *E. coli*) by isothermal titration and differential scanning calorimetry to characterize the specific interactions between SVN/SD1 and gp120 as well as to high-mannose oligosaccharides. SVN bound with moderate affinity (K_d_ = 1.5 µM) to recombinant gp120, with 2.5-fold weaker affinity to nonamannoside (K_d_ of 3.9 µM), and with tenfold weaker affinity to tetramannoside (13.8 µM). The melting temperature (T_m_) of full-size SVN was 59.1 °C and the enthalpy of unfolding (ΔH_unf_) was 16.4 kcal/mol, but the T_m_ fell when SVN bound to nonamannoside (56.5 °C) and twice as much energy was required for unfolding (ΔH_unf_ = 33.5 kcal/mol). Interestingly, binding to tetramannoside destabilized the structure of SD1 (ΔT_m_ ~ 11.5 °C) and doubled the enthalpy of unfolding, suggesting a dimerization event. The similar melting phenomenon shared by SVN and SD1 in the presence of oligomannose confirmed their conserved oligosaccharide-binding mechanisms. SD1 expressed in transgenic rice was able to neutralize HIV-1 in vitro. SD1 expressed in rice, therefore, is suitable as a microbicide component.

## Introduction

Lectins are carbohydrate-binding proteins that bind reversibly to specific glycan structures (Mitchell et al. 2017; Singh et al. 2017). Many lectins recognize the *N*-linked glycans present on viral glycoproteins (François and Balzarini 2012; Kumar et al. 2012) and can, therefore, prevent viral proteins from interacting with receptors on the target cell surface, blocking the uptake of viral particles and interrupting the infection cycle (Balzarini 2006; Botos and Wlodawer 2005).

A recognized target of antiviral lectins is the human immunodeficiency virus (HIV), which has thus far infected ~ 76 million people,  ~ 38 million of whom are still living with the virus today (WHO 2020). HIV-1 is the most common and virulent type of HIV. It enters susceptible cells when the viral surface glycoprotein gp120 interacts with the receptor CD4 on the surface of lymphocytes. This is followed by the binding of co-receptors (CCR5 or CXCR4), allowing the transmembrane glycoprotein gp41 to induce membrane fusion (Steckbeck et al. 2013). Molecules such as lectins, that bind gp120 and/or gp41, can, therefore, act as HIV-1 entry inhibitors and may be suitable as topical microbicides, representing a subset of pre-exposure prophylaxis strategies (Xion et al. 2006; Vamvaka et al. 2018). Lectins with the potential to act as HIV-1 entry inhibitors include cyanovirin-N (CV-N) from the cyanobacterium *Nostoc ellipsosporum* (Boyd et al. 1997) and griffithsin (GRFT) from the red alga *Griffithsia sp.* (Mori et al. 2005), both of which bind oligomannose structures on gp120 and can neutralize the virus before target cell uptake (Mori et al. 2002). Both lectins have been investigated as topical microbicides to prevent HIV-1 transmission (Mori et al. 2005; O’Keefe et al. 2009; Moulaei et al. 2010; Vamvaka et al. 2016b, 2016c) and GRFT has recently entered human clinical trials (ClinicalTrials.gov identifier: NCT02875119 and NCT04032717). Another microbicide candidate is scytovirin (SVN) from the cyanobacterium *Scytonema varium* (Bokesch et al. 2003). The 95-amino-acid SVN polypeptide has a molecular weight of 9.7 kDa and contains two structural domains, namely SD1 (residues 1–48) and SD2 (residues 49–95), linked by five intra-chain disulfide bonds (McFeeters et al. 2007; Moulaei et al. 2007). SVN can neutralize HIV-1 by binding to gp120, gp160 and gp41. SD1 has demonstrated similar activity to full-length SVN, whereas SD2 is less potent than the full-length SVN and SD1 (Xiong et al. 2006). The domains have different affinities for HIV-1 and bind to carbohydrate ligands independently. Native SVN is active against both laboratory strains and primary isolates of HIV-1, the latter providing a more faithful representation of the inoculum that humans encounter during HIV-1 transmission, with EC_50_ values ranging from 0.3 to 22 nM (Bokesch et al. 2003).

To help prevent the spread of HIV, viral entry inhibitors such as broadly neutralizing monoclonal antibodies and lectins have been formulated as microbicides for pre-exposure prophylaxis, and have been tested in vitro, in animal studies, and in human clinical trials (O’Keefe et al. 2010; Ramessar et al. 2010; McCoy and Weiss 2013; Bar et al. 2016). Entry inhibitors can be produced as recombinant proteins by fermentation in mammalian cells (antibodies) or microbial systems (lectins), but these platforms are too expensive for the large-scale and low-margin production campaigns needed to manufacture microbicides for target populations that are disproportionately found in countries with small budgets for public health (Ma et al. 2003; Stoger et al. 2005; Mir‐Artigues et al. 2019). The demand is created by the large size of the at-risk population, the frequency of microbicide application, and the large doses required for efficacy (Ramessar et al. 2008; Ma et al. 2013; Sabalza et al. 2014). Transgenic plants can address these drawbacks, allowing inexpensive microbicide production (Capell et al. 2020; Lobato Gomez et al. 2021; He et al. 2021). In the context of microbicides, transgenic cereals offer additional advantages because lectins and/or antibodies can be produced locally and stored/transported as dry seed (obviating the need for a cold chain), and the products can be administered directly as crude extracts prior to intercourse, thus eliminating the costs of downstream processing (Ramessar et al. 2010; Stoger et al. 2005; Sabalza et al. 2013). However, before transgenic plants can be used for the large-scale production of microbicidal lectins, it is necessary to ensure the recombinant proteins remain functional by characterizing their physicochemical interactions.

Here we investigated the suitability of the lectin SD1 as a plant-derived microbicide component. Using isothermal titration calorimetry (ITC), that measures the heat of association for binding interactions between two macromolecules, and by differential scanning calorimetry (DSC), which measures the heat change associated with the thermal denaturation of a molecule when heated at a constant rate, we compared the thermodynamic behavior of SD1 to full-size SVN produced in *E. coli*. We analyzed the binding of SD1 and SVN to HIV-1 envelope glycoprotein gp120 as well as individual high-mannose oligosaccharides. The crude plant extract containing SD1 was also tested for HIV-binding activity and its ability to prevent HIV-1 infection in vitro using the TRO11 pseudovirus, which covers the diversity of most circulating strains of HIV-1 group M subtype B, as well as primary isolates of HIV-1.

## Materials and methods

### Genetic constructs and transgenic plants

The *S. varium SD1* gene in vector pET-28a ( +) (National Cancer Institute, Frederick, MD, USA) was amplified by PCR in a 25 µL reaction containing 0.125 µL GoTaq G2 Flexi DNA polymerase (5 U/µL) in 1 × Green GoTaq Reaction Buffer (Promega, Madison, WI, USA), 0.5 µL (10 µM) forward primer 5′-GGA TCC ATG AGC GAT AAA ATT ATT C-3′ (BamHI restriction site underlined) and reverse primer 5′-GAA TTC GCA GCC GGA TCT CAG TGG TG-3′ (EcoRI restriction site underlined). The reaction was heated to 95 °C for 2 min followed by 35 cycles of 95 °C for 45 s, 60 °C for 45 s and 72 °C for 5 min and a final extension step at 72 °C for 10 min. The product was transferred to the shuttle vector pGEM-T easy (Promega) and introduced into competent *Escherichia coli* cells, which were incubated overnight at 37 °C with ampicillin for selection. Plasmid DNA from positive colonies was verified by sequencing (StabVida, Caparica, Portugal). Plasmid DNA was digested with BamHI and EcoRI (Promega) and the *SD1* gene was transferred to vector pRP5 containing the endosperm-specific rice prolamin promoter (Naqvi et al. 2009) and the nos terminator.

### Generation of transgenic rice plants and confirmation of SD1 gene expression

Seven-day-old mature rice zygotic embryos (*Oryza sativa* cv. Nipponbare) were transferred to Murashige and Skoog (MS) osmotic medium (4.4 g/L MS salts supplemented with 0.3 g/L casein hydrolysate, 0.5 g/L proline, 72.8 g/L mannitol and 30 g/L sucrose) 4 h before bombardment with 10 mg gold particles coated with the construct carrying the *SD1* transgene and second construct carrying the selectable marker *hpt*. The embryos were returned to MS osmotic medium for 12 h before selection on standard MS medium (4.4 g/L MS salts, 0.3 g/L casein, 0.5 g/L proline and 30 g/L sucrose) supplemented with 50 mg/L hygromycin and 2.5 mg/L 2,4-dichlorophenoxyacetic acid in the dark for 2–3 weeks. Transgenic plantlets were regenerated and hardened off in soil. Plants were grown in the greenhouse or growth chamber at 28/20 °C day/night temperature with a 10 h photoperiod and 60–90% relative humidity for the first 50 days, followed by maintenance at 21/18 °C day/night temperature with a 16 h photoperiod thereafter in a growth chamber.

To confirm and quantify *SD1* transgene expression in transgenic plants, total RNA was extracted from 21-day-old immature seeds using the LiCl method and cDNA was prepared using the QuantiTect reverse transcription kit (Qiagen, USA). Each 25 μl reaction contained 10 ng cDNA, 1 × SsoAdvanced Universal SYBR Green Supermix (Bio-Rad Laboratories, Hercules, CA, USA), 0.2 μM forward primer (5′-ACG GAT GTA CTC AAA GCG GAC-3′) and 0.2 μM reverse primer (5′-TAT GGC ATC CGT GGT ATC CCG-3′). Quantitative PCR was carried out by heating the reaction to 95 °C for 3 min followed by 39 cycles of 95 °C for 10 s, 59 °C for 30 s and 72 °C for 20 s and a final step at 95 °C for 10 s using CFX96 system (Bio-Rad Laboratories). Amplification specificity was confirmed by melt curve analysis on the final PCR products in the temperature range 50–90 °C with fluorescence acquired after each 0.5 °C increment. Values represent the mean of three technical replicates. The relative expression was calculated using the 2-ΔCt formula, where ΔCt is the threshold cycle difference between the *actin* and *SD1* genes.

### Quantification of SD1 protein and in vitro analysis of binding to gp120

The presence of SD1 in transgenic rice endosperm was confirmed by enzyme-linked immunosorbent assay (ELISA). Mature rice seeds (25 seeds per sample, equivalent to 375 mg) were ground in three volumes of phosphate-buffered saline (PBS, pH 7.4) and centrifuged twice (13,000×*g*, 10 min, 4 °C). ELISA plates were coated with 50 ng/well of recombinant gp120 from HIV-1 strain IIIB provided by the MRC Centralized Facility for AIDS Reagents (Potters Bar, UK) overnight at 4 °C. The plates were then washed with PBS and incubated for 2 h at room temperature with blocking solution comprising 1% bovine serum albumin (BSA; Sigma–Aldrich, Madrid, Spain) in PBS containing 0.1% Tween-20 (PBST). SD1 was detected in serial dilutions of seed extract using a primary rabbit anti-SVN polyclonal antiserum (National Cancer Institute, Bethesda, MD, USA) diluted 1:2000 and a secondary horseradish peroxidase (HRP)-conjugated anti-rabbit IgG antibody (Sigma–Aldrich) diluted 1:10,000. HRP activity was detected by staining with 3,3′,5,5′-tetramethylbenzidine (TMB) substrate (Thermo Fisher Scientific, Waltham, MA, USA). The reaction was stopped with 0.16 M H_2_SO_4_, and absorbance was measured at 450 nm. Recombinant SD1 purified from *E. coli* (Xion et al. 2006) was used as a positive control and wild-type rice extract and 1% BSA in PBS as negative controls. The yield of SD1 in rice seeds (ng/g dry seed) was calculated as the product of the OD_450_ value, taking the rice extract dilution factors into consideration and the volume of buffer used for the protein extraction, divided by the weight of the seeds. Known concentrations of SD1 purified from *E. coli* were used to perform the standard curve. The specific antigen-binding activity of SD1 was determined using the same procedure.

### Isothermal titration calorimetry

ITC was carried out using a VP-ITC device (Malvern Panalytical, Malvern, UK). For the SVN:gp120 titration experiments, 102 µM *E. coli*-produced SVN (Xion et al. 2006) was titrated into a calorimetry cell containing 1.6 µM gp120. For the sugar titration experiments, 2.4 mM nonamannoside or 1 mM tetramannoside was titrated into a calorimetry cell containing 102 µM SVN. Purified oligosaccharides were either purchased from Glyko Inc. (Novato, CA) or were the kind gift of Dr. Peter Seeberger (Max Planck Institute) and were synthesized as previously described (Ratner et al. 2002). In a typical experiment, 5 µL aliquots of titrant were injected into a rapidly mixing (300 rpm) solution in the calorimetry cell (volume = 1.4426 mL) with a total of 55 injections during the experiment. Controls were prepared with identical amounts of titrant injected into a protein-free buffer, and control values were subtracted from the results of the other experiments. Titrations were carried out at 30 °C in 10 mM sodium phosphate buffer (pH 7.4) containing 3 mM sodium azide. The isotherm, corrected for dilution/buffer effects, was fitted to a nonlinear least squares curve-fitting model (for a 1-set of identical sites) using Microcal Origin v7.0 (OriginLab, Northampton, MA, USA). We extracted values for enthalpy, binding affinity and stoichiometry from the binding curve, and the free energy and entropy of interaction were calculated using Eqs. (1) and (2):

1 $$\Delta {\text{G }} = \, - {\text{RTlnK}}_{{\text{a}}}$$

2 $$\Delta {\text{G }} = \, \Delta {\text{H }} - {\text{ T}}\Delta {\text{S}}$$

where *∆G* is the change in Gibbs free energy, *R* is the gas constant (~ 1.987 cal/mol K), *T* is the absolute temperature (303 K), *K*_a_ is the equilibrium constant, *∆H* is the change in enthalpy and *∆S* is the change in entropy.

### Differential scanning calorimetry

DSC was carried out using a VP-DSC device (Malvern Instruments). In a typical experiment, the concentration of *E. coli*-produced SVN or SD1 (Xion et al. 2006) was adjusted to 20 µM and was prepared in a total volume of 1.0 mL with or without sugars in 10 mM sodium phosphate buffer (pH 7.4) containing 3 mM sodium azide. Before adding the samples, the degassed buffer solutions were first placed in the sample and reference cells. Following the first up scan (10–95 °C) and during the first down scan (at 25 °C), the sample cell was emptied and filled with a degassed SVN (or SD1) solution. The sample and cell compartments were capped with adjusted positive pressure (30 psi) and allowed to progress through eight alternating up and down scans at 60 °C/h with a filter period of 16 s. The completed sample thermogram was corrected for buffer effects (subtracting the first up scan) followed by normalized correction of the absolute baseline and cubic approximation of the pre-transition (structured) and post-transition (unstructured) regions of the thermogram. The baseline-corrected thermogram was fitted to a non-2-state unfolding model and the fitting error was evaluated for single versus multiple unfolding units. From this model-fitting, values for melting temperature (T_m_) and enthalpy of unfolding (*∆*Hcal) were extrapolated for each transition. The reversibility of melting was assessed by superimposing the repeated up-scans. For compound experiments, the post-titrated solutions of SVN (or SD1) with sugars were adjusted to a final protein concentration of 20 µM. Similar parameters and procedures were used to assess changes to T_m_ and the enthalpy of unfolding.

### HIV-neutralization assays

HIV-1 pseudoviruses BaL (laboratory isolate), TRO11 and AC10 (primary isolates) were generated by the co-transfection of HEK293T cells with HIV-1 Env protein-expressing plasmids (NIH AIDS Reagent Program, Manassas, VA, USA) and the PSG3 vector as previously described (Sánchez-Palomino et al. 2011). The supernatants were harvested 24 h post-transfection and were passed through a 0.45 μm filter and stored at − 80 °C. Viral titration and neutralization were tested using a standard TZM-bl assay. Briefly, serially diluted samples were pre-incubated in duplicate with 200 TCID_50_ of pseudovirus stock for 1 h at 37 °C in 96-well culture plates. We then added 10,000 TZM-bl luciferase-reporter cells to each well. The plates were incubated at 37 °C in a 5% CO_2_ atmosphere for 48 h in Dulbecco’s modified Eagle’s medium (DMEM; Thermo Fisher Scientific) supplemented with 10% fetal bovine serum (FBS) and 30 μg/ml dextran (Sigma–Aldrich). The luciferase substrate Britelite Plus (PerkinElmer, Waltham, MA, USA) was used for the readout. Percent neutralization was determined by calculating the difference in average relative light units (RLUs) between virus control (cells + virus) and test wells (cells + sample + virus). Neutralizing titers are expressed as the concentration of total protein or SD1 necessary to reduce the RLUs by 50% (IC_50_). All data were fitted to an inhibitor vs response (three parameters) model using GraphPad Prism v6 (GraphPad Software, San Diego, CA, USA).

## Results

### Expression of SD1 in transgenic plants and gp120-binding activity in vitro

**Fig. 1:**
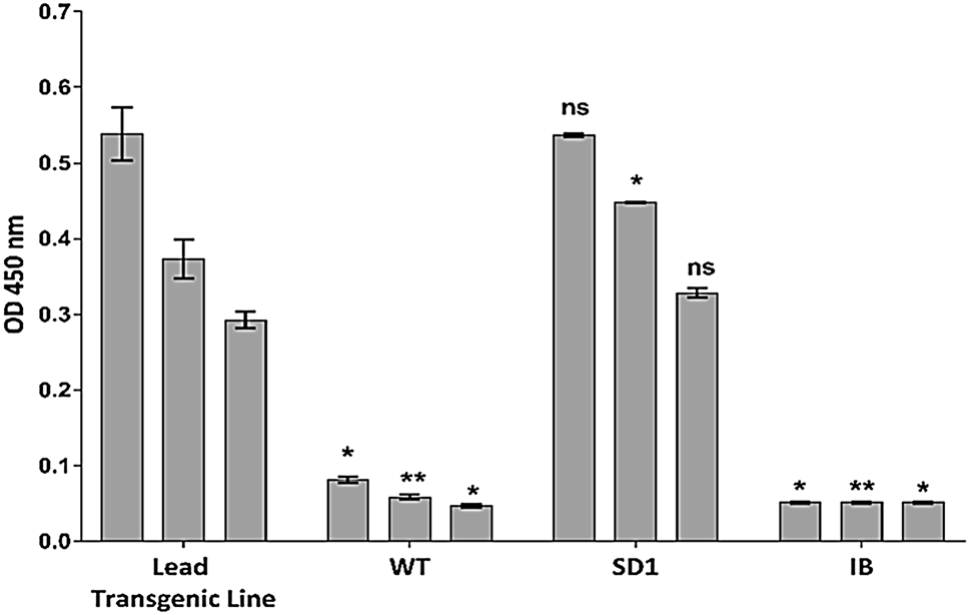
Rice endosperm extracts (containing SD1) bind to gp120. Plates were coated with gp120 (50 ng/well). SD1 was detected with a primary rabbit anti-SVN polyclonal antiserum. Lead Transgenic Line: starting concentration corresponds to 0.5 dilution of the original endosperm extract. WT: starting concentration corresponds to 0.5 dilution of the original endosperm extract. SD1: purified from *E. coli* and used as a positive control (starting concentration was 4 ng/mL). IB: 1% BSA in PBST. A fourfold dilution series were performed. Data are means ± standard errors (*n* = 3). Turkey test (ANOVA) with a *P* ≤ 0.05 was performed. Asterisks (*) indicate significant differences and ns indicates no significant differences comparing to each dilution of the Lead Transgenic Line

Mature seed-derived rice embryos were co-transformed with a construct containing the *SD1* coding sequence under the control of an endosperm-specific promoter, plus a second construct containing the selectable marker *hpt* (Christou et al. 1991). Embryo-derived callus was selected on hygromycin-supplemented medium and independent transformants were regenerated and transferred to the greenhouse. Total RNA extracted from T1 seeds was reverse transcribed using SD1-specific primers and amplified by qPCR to confirm transgene expression. The presence of correctly assembled SD1 protein in the endosperm was confirmed by ELISA and the yield was determined by calculating the concentrations from titration curves based on positive controls spiked with known amounts of SD1 produced in *E. coli* and non-transformed rice endosperm as a negative control. We selected the line which accumulated SD1 at the highest level (26 ng/g dry seed weight) for subsequent experiments. Crude endosperm extracts from this transgenic line were tested for their ability to bind HIV gp120IIIB in vitro, using SD1 produced in *E. coli* as positive control and wild-type rice endosperm extracts as a negative control. We observed similar concentration-dependent gp120 binding in the positive control and in the extracts of the transgenic seeds (Fig. 1).

### Thermodynamic analysis of *E. coli*-produced SVN and SD1 binding interactions

**Fig. 2:**
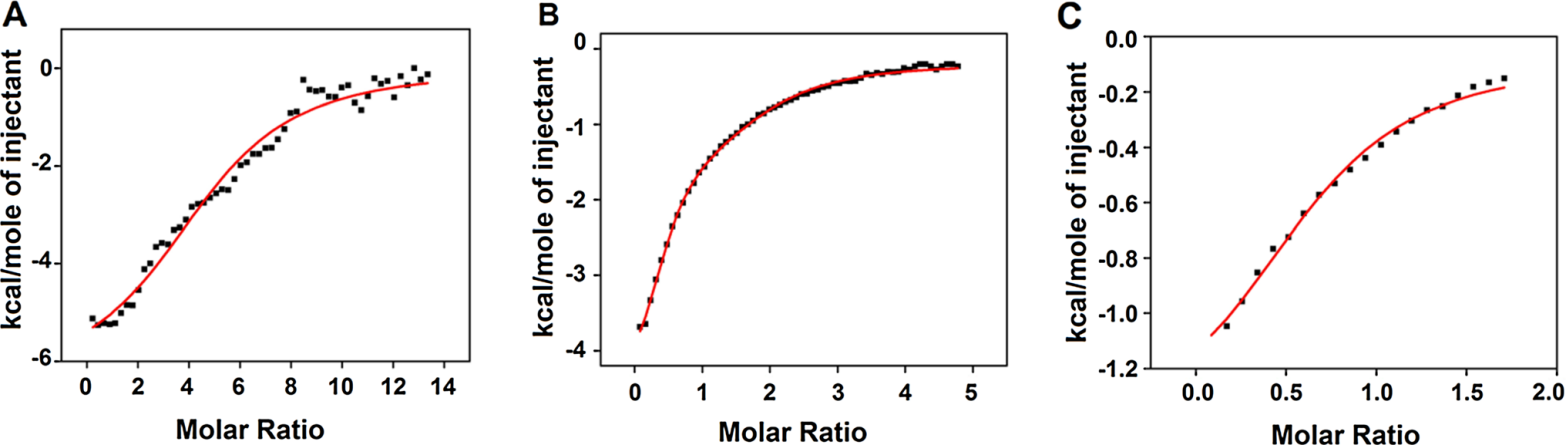
SVN binding kinetics characterized by isothermal titration calorimetry. **A** Binding of SVN to gp120. **B** Binding of nonamannoside to SVN. **C** Binding of tetramannoside to SVN

The ITC isotherms (Fig. 2) were fit to a nonlinear least squares curve-fitting model for identical binding sites. The values for enthalpy, binding affinity were extracted directly from this curve fitted model (shown in red, Fig. 2) and the free energy and entropy of interaction were back calculated using Eqs. 1, 2 (“Materials and Methods”). All thermodynamic parameters extracted from this curve-fitting analysis are listed in Table 1. The mid-point of the transitions (Fig. 2) extrapolated to the X-axis indicates the stoichiometry of the interaction between SVN and its binding partners. As can be seen in Table 1, the binding between SVN and gp120 was driven by both favorable enthalpic and entropic contributions (negative ΔH value and positive ΔS value, respectively) and resulted in a moderate binding affinity of K_d_ = 1.63 µM. The stoichiometry of the interaction was 5:1, five molecules of SVN binding to a single molecule of gp120 (Fig. 2A). In contrast, SVN bound to oligomannosides with 1:1 stoichiometry (Fig. 2B and C) and the binding was predominantly driven entropically (positive ΔS value; Table 1). As expected, the enthalpy of binding was significantly lower when SVN bound to the oligosaccharides rather than gp120, presumably because fewer surface contacts are available on the smaller molecules. Accordingly, the enthalpy–entropy compensation reduced the free energies of binding (ΔG), resulting in a ~ 2.5-fold lower affinity for nonamannoside (K_d_ = 4.0 µM) and a ~ tenfold lower affinity for tetramannoside (K_d_ = 17.8 µM) (Table 1).

**Table 1:** Thermodynamic binding parameters of SVN as determined by isothermal titration calorimetry

Interaction	K_d_ µM	ΔH kcal/mol	ΔS cal/mol K	ΔG kcal/mol
SVN + gp120	1.63 ± 0.22	–6.45 ± 0.27	5.18 ± 0.89	–8.02 ± 0.08
Nonamannoside + SVN	3.99 ± 0.93	–4.71 ± 0.10	9.13 ± 0.45	–7.48 ± 0.14
Tetramannoside + SVN	17.80 ± 2.75	–1.98 ± 0.12	15.19 ± 0.39	–6.58 ± 0.09

K_d_ = binding affinity reported as the equilibrium dissociation constant, which is used to evaluate and rank the strength of bimolecular interactions. ΔH = enthalpy change as the heat of a process at constant pressure. ΔS = change in entropy determined using the equation ΔG = ΔH − TΔS for finite variations at constant temperature. ΔG = change in the free energy of a system as it transitions from an initial to a final state

**Fig. 3:**
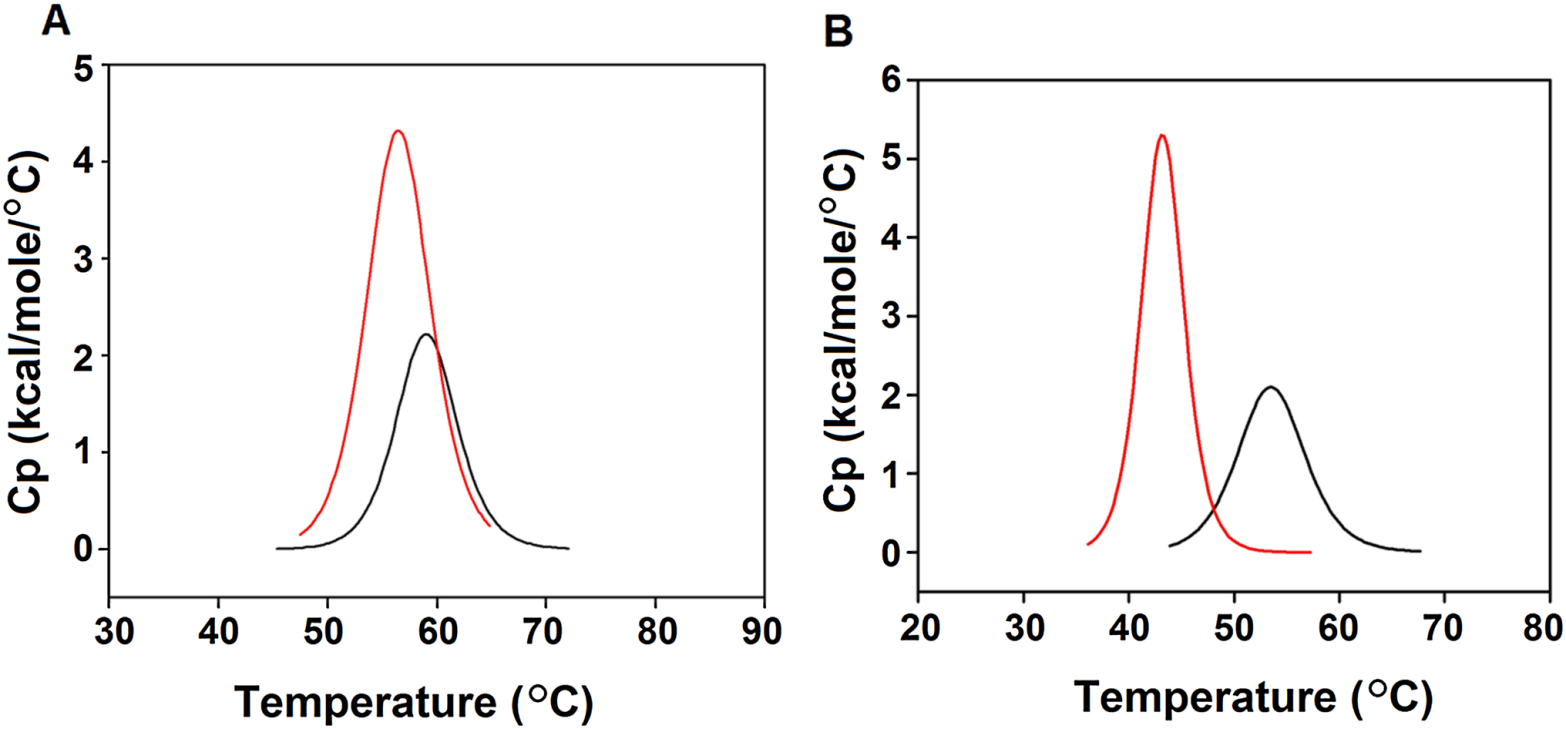
SVN stability characterized by differential scanning calorimetry. **A** The melting of SVN in the absence (black) and in the presence of nonamannoside (red). **B** The melting of SD1 in the absence (black) and in the presence of tetramannoside (red) (color figure online)

DSC results showed SVN melted at T_m_ = 59.1 °C and had an enthalpy of unfolding (ΔH) of 16.4 kcal/mol (Table 2). In the presence of nonamannoside, the oligosaccharide–protein complex melted at a lower temperature (T_m_ = 56.5 °C) but required twice as much energy (ΔH = 33.5 kcal/mol) for unfolding (Fig. 2B and C). Similarly, DSC experiments with SD1 showed that binding to tetramannoside destabilized the SD1 structure and caused a twofold increase in the enthalpy of unfolding (Fig. 3). These data suggest that the tertiary structures of SVN and SD1 were destabilized (lower T_m_) when binding the oligosaccharides, favoring the dimerization of the oligosaccharide–protein complexes. The ability of SVN and SD1 to oligomerize via crosslinking with sugars is consistent with other anti-HIV lectins such as CV-N (Shenoy et al. 2002).

**Table 2:** Thermodynamic parameters of SVN/SD1 melting as determined by differential scanning calorimetry

	T_m_ °C	ΔH kcal/mol
SVN	59.05 ± 0.04	16.40 ± 0.21
Nonamannoside + SVN	56.50 ± 0.05	33.50 ± 0.51
SD1	53.55 ± 0.07	17.80 ± 0.32
Tetramannoside + SD1	42.02 ± 0.08	28.50 ± 0.90

### Neutralization of HIV pseudoviruses in vitro by crude rice endosperm extracts containing SD1

**Fig. 4:**
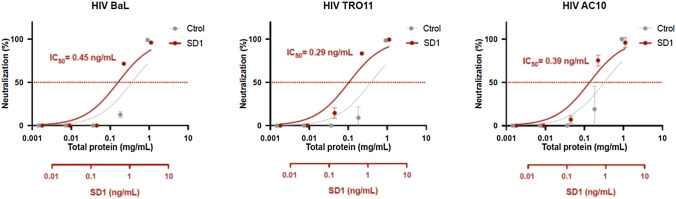
Neutralization activity of the extract from Lead Transgenic Line expressing SD1 (SD1) and wild-type rice extract (Ctrol) in a pseudovirus assay using BaL, TRO11 and AC10 isolates. Extracts were normalized by the total amount of protein (*x*-axis). The calculated concentration of SD1 is also shown in the additional red *x*-axis. Apparent SD1 IC_50_ values are indicated for each pseudovirus. Data are means ± standard errors (*n* = 2). The experiment was carried out twice with the same results. Notice that some error bars are too small to be visible at this scale

The activity of extracts from the transgenic line was tested in HIV infectivity and neutralization assays with three HIV pseudoviruses expressing the envelope glycoprotein of the laboratory adapted isolate BaL or the primary isolates TRO11 and AC10. Wild-type rice endosperm extracts were used as a negative control. The extract of the transgenic seeds was able to neutralize all three pseudoviruses with IC_50_ values ranging from 0.10 to 0.16 mg protein/mL, corresponding to apparent IC_50_ values for SD1 ranging from 0.29 to 0.45 ng/mL (Fig. 4). Interestingly, the wild-type rice extract also demonstrated significant neutralization activity at the highest concentration tested, with IC_50_ values ranging from 0.32 to 0.38 mg protein/mL, indicating that components of the rice endosperm can reduce virus infectivity independently of the lectin (Fig. 4).

## Discussion

The biological function of many lectins is associated with self-recognition and cell adhesion in multicellular organisms. Lectins from natural sources have been shown to bind to the *N*-linked glycans on the surface of many enveloped viruses (Goldstein and Poretz 1986; François and Balzarini 2012; Kumar et al. 2012) resulting in the disruption of interactions between proteins on the viral envelope and their cellular receptors (Balzarini 2006; Botos and Wlodawer 2005; Sacchettini et al. 2001; Shenoy et al. 2002). This characteristic makes them useful as broad-spectrum antiviral agents, particularly for use in pre-exposure prophylaxis (O’Keefe et al. 2010; Ramessar et al. 2010; McCoy and Weiss 2013; Bar et al. 2016). For example, SVN from the cyanobacterium *S. varium* (Bokesch et al. 2003) binds to mannose-rich oligosaccharides on the envelope glycoproteins of several viruses, including Ebola (Garrison et al. 2014), dengue and other flaviviruses with the same glycoprotein pattern (Siqueira et al. 2017), hepatitis C virus (Takebe et al. 2013), and HIV-1 (Xiong et al. 2006).

SVN has been proposed as a candidate HIV entry inhibitor for therapeutic and prophylactic applications (McFeeters et al. 2007). It has been expressed for this purpose in *E. coli* (Xiong et al. 2006) and *Lactobacillus plantarum* (Janahi et al. 2018), with both bacterial recombinant proteins able to bind HIV-1 gp160 but the version produced in *E. coli* demonstrating marginally higher potency against HIV-1 in a TZM-bl cell assay: ~ 89% reduction in infectivity after 48 h compared to ~ 86% for the version produced in *L. plantarum* (Janahi et al. 2018). SVN is particularly active against HIV because it can bind to multiple sites on gp120 (Bokesch et al. 2003; Siqueira et al. 2017). Specifically, SVN binds to α1,2-α1,2-α1,6-linked tetramannoside (Adams et al. 2004) as well larger oligosaccharides such as Man-8 and Man-9 on gp120 and gp41 (Xiong et al. 2006). The ability of SVN to bind multiple sites reflects the presence of two domains (SD1 and SD2), with partially conserved tertiary structures, which can each bind independently to oligosaccharides. The combination of multisite and multivalent binding by SVN increases its potency. When these domains were expressed separately in *E. coli*, SD1 showed equivalent antiviral activity to full-size SVN whereas SD2 bound to gp120 with 50% lower affinity (Xiong et al. 2006), which may reflect the flipped orientation of the middle-turn regions within the individual carbohydrate-binding domains (McFeeters et al. 2007).

Transgenic plants offer an inexpensive and scalable production platform for lectins as a step toward the development of microbicidal cocktails based on plant extracts (Ma et al. 2003; Stoger et al. 2005; Ramessar et al. 2008; Lobato Gomez et al. 2021). The seeds of cereals such as rice are particularly suitable because they have “generally regarded as safe” status, allowing the direct application of extracts to the mucosal surface, and recombinant proteins in dry seeds remain stable for many years, thus addressing the limited availability of cold chains in developing countries (Daniell et al. 2001; Ramessar et al. 2008; Ma et al. 2013; Arcalis et al. 2014). We have previously used rice to express the HIV-neutralizing antibody 2G12 (Vamvaka et al. 2016a) and the lectins GRFT (Vamvaka et al. 2016b) and CV-N (Vamvaka et al. 2016c), as well as all three of these components simultaneously (Vamvaka et al. 2018). We found that the recombinant proteins retained their physicochemical properties and biological activity when compared to positive controls produced in *E. coli*. Before using transgenic plants for the large-scale production of SVN and/or its smaller congener SD1, it was necessary to conduct similar tests to ensure that the recombinant proteins can still bind with high affinity to HIV glycoproteins and neutralize the virus in vitro.

We compared the biological activity of recombinant SD1 proteins produced in *E. coli* and rice by testing their ability to bind gp120 in vitro and found that both versions showed comparable binding activity. Rice-produced SD1 showed the same concentration-depending affinity for gp120 as *E. coli* SD1 (the starting concentration of SD1 protein tested by ELISA (Fig. 1) was 4 ng/mL in both cases) indicating that the rice-produced protein has the same oligosaccharide-dependent binding properties as the bacterial-expressed protein (Xiong et al. 2006). We probed the physicochemical basis of this binding activity in ITC and DSC experiments to gain more insight into the specific interactions between *E. coli*-produced SVN/SD1 and HIV envelope glycoproteins and/or high-mannose oligosaccharides. ITC revealed a K_d_ value of 1.5 µM for SVN binding to gp120, and the post-titrated solution was clear, ruling out the presence of insoluble protein–protein aggregates. The binding of SVN to gp120 resulted in a 5:1 stoichiometry, which was the same as previously reported for CV-N (O’Keefe et al. 2000). This suggests that both lectins may recognize similar or identical surface contacts on gp120. Previous studies using microarrays indicated that SVN binds terminal α1,2-mannose on the D3 arm of gp120 and can, therefore, recognize Man-9, Man-8 and even a tetramannoside structure mimicking the D3 arms of Man-9 (Ratner and Seeberger 2007), but not Man-7, which lacks a terminal α1,2-mannose. Accordingly, we characterized the binding of SVN to tetramannoside and nonamannoside (similar to Man-9 but missing the core GlcNAc residues) by ITC. SVN bound to both structures with 1:1 stoichiometry but with a significant difference in affinity between the nonamannoside (K_d_ = 4 µM) and tetramannoside (K_d_ = 18 µM). SVN binding to nonamannoside was enthalpically more favorable (ΔH = –5.3 kcal/mol) than tetramannoside (ΔH = –1.9 kcal/mol), suggesting the presence of more polar/electrostatic, van der Waals and hydrogen bond contacts on nonamannoside compared to the smaller sugar structure. In addition, nonamannoside is a branched sugar and as such can engage in multivalent interactions with SVN via its multiple α1,2-mannose termini, this may account for the stronger binding compared to tetramannoside. Interestingly, a baseline disturbance was noted in the nonamannoside-SVN ITC experiment, suggesting that soluble high-molecular-weight complexes may have formed during the titration. It is also likely that SD1 and SD2 engage in multisite binding, which combined with the multivalency of nonamannoside can explain the higher affinity for this oligosaccharide while maintaining an overall 1:1 stoichiometry. However, if such higher-order structures were formed, they did not persist, hence the absence of aggregation or cloudiness in the post-titrated solution. The difference in carbohydrate-binding strength between SD1/SD2 and the domains of CV-N may be the reason SVN cannot lock in the higher-order cross-linked structures with branched oligosaccharides. This may be the real reason for its inability to achieve the type of potent irreversible binding and inactivation of gp120 that has been observed for CV-N (Xiong et al. 2006).

DSC experiments indicated that SVN melted at a moderate temperature (T_m_ = 59.1 °C) and that the enthalpy of unfolding (ΔH) was 16.4 kcal/mol, as would be expected for a 10-kDa protein. In the presence of nonamannoside, the oligosaccharide–protein complex melted at a lower temperature (T_m_ = 56.5 °C), but required twice as much energy (ΔH = 33.5 kcal/mol) for unfolding. The lower melting temperature suggests that at least some part of the tertiary structure of SVN was destabilized in the presence of the sugar and that either the destabilized structure had exposed hydrophobic surface patches that allowed for potential protein–protein dimerization contacts, or that the sugar itself facilitated crosslinking during dimerization. A similar phenomenon was observed in the DSC experiments with SD1, where binding to tetramannoside destabilized the SD1 structure (ΔTm ~ 11.5 °C) and resulted in a twofold increase in the enthalpy of unfolding. Additional structural studies may resolve the nature of the destabilization mediated by the oligosaccharide binding and the resulting dimerization event. Such studies may also help to determine whether the dimeric structure is entirely composed of protein or is bridged by an oligosaccharide. Our DSC results show that the full-length SVN and its congener SD1 both melt in a similar manner upon binding to oligomannose sugars, suggesting that they may share an analogous mode of interaction with the sugars on gp120 and may, therefore, demonstrate similar HIV-neutralizing activities.

Accordingly, we tested the neutralization activity of rice-expressed SD1 in an HIV-1 pseudovirus assay based on three different Env glycoprotein variants, a laboratory adapted strain and two primary isolates representing the diversity of most circulating HIV-1 strains of group M subtype B (Hemelaar et al. 2006). SD1 from rice exhibited nanomolar activity against HIV and protected cells from infection (IC_50_ = 0.29 to 0.45 ng/mL). Interestingly, the wild-type crude extract also showed modest HIV-neutralizing activity even though there was no significant binding to HIV-1 gp120, with an IC_50_ 2.4–3.8 times higher than the one obtained for the extract of the transgenic seeds (Fig. 1), suggesting that virus neutralization may be an indirect effect perhaps involving interactions with the host cell surface or molecular crowding (Vamvaka et al. 2018).

In conclusion, our data suggest that rice-derived SD1 retains its physicochemical and biological properties and is, therefore, a promising candidate microbicide for pre-exposure HIV-1 prophylaxis. SD1 has fewer disulfide bonds than SVN and is smaller and, therefore, probably less immunogenic. Its low nanomolar activity and physical stability are major advantages for a microbicide component (Xiong et al. 2006). The functionality of SD1 in the crude rice extract and the minimal processing and purification steps required during manufacturing will reduce production costs significantly compared to traditional cell-based platforms.
